# Effectiveness of intravenous immunoglobulin alone and intravenous immunoglobulin combined with high-dose aspirin in the acute stage of Kawasaki disease: study protocol for a randomized controlled trial

**DOI:** 10.1186/s12887-018-1180-1

**Published:** 2018-06-22

**Authors:** Ho-Chang Kuo, Mindy Ming-Huey Guo, Mao-Hung Lo, Kai-Sheng Hsieh, Ying-Hsien Huang

**Affiliations:** 1 Department of Pediatrics, Kaohsiung Chang Gung Memorial Hospital and Chang Gung University College of Medicine, #123 Da-Pei Road, Niaosong District, Kaohsiung, 83301 Taiwan; 2 Kawasaki Disease Center, Kaohsiung Chang Gung Memorial Hospital, Kaohsiung, Taiwan; 3 Department of Pediatrics, Chiayi Chang Gung Memorial Hospital, Kaohsiung, Taiwan

**Keywords:** Coronary artery lesions, Intravenous immunoglobulin, Kawasaki disease, Randomized clinical trial

## Abstract

**Background:**

Kawasaki disease (KD) is an acute febrile systemic vasculitis most commonly seen in children under 5 years old. High-dose aspirin is often administered, but the duration of such treatment varies. Many centers reduce the aspirin dose once the patient is afebrile, even before treating said patient with intravenous immunoglobulin (IVIG). However, a randomized controlled trial regarding high-dose aspirin in the acute stage of KD has not previously been carried out.

**Methods/design:**

This trial has been designed as a multi-center, prospective, randomized controlled, evaluator-blinded trial with two parallel groups to determine whether IVIG alone as the primary therapy in acute-stage KD is as effective as IVIG combined with high-dose aspirin therapy. The primary endpoint is defined as coronary artery lesion (CAL) formation at 6–8 weeks. Patients meeting the eligibility criteria are randomly assigned (1:1) to a test group (that receives only IVIG) or a standard group (that receives IVIG plus high-dose aspirin). This clinical trial is conducted at three medical centers in Taiwan.

**Discussion:**

Since high-dose aspirin has significant anti-inflammatory and anti-platelet functions, it does not appear to affect disease outcomes. Furthermore, it can decrease hemoglobin levels. Therefore, we have initiated this randomized controlled trial to evaluate the necessity of high-dose aspirin in the acute stage of KD.

**Trial Registration:**

Kawasaki Disease Center, Kaohsiung Chang Gung Memorial Hospital, Taiwan. ClinicalTrials.gov Identifier: NCT02951234. Release Date: November 3, 2016.

## Background

Kawasaki disease (KD) is an acute febrile systemic vasculitis that was first described in 1967 by Kawasaki et al. [1]. Although its etiology is still unknown, KD is the leading etiology of acquired heart diseases in children in developed countries [2–4]. It affects children around the world, mainly those less than 5 years old. In Japan, Taiwan, and Korea, the incidence ranges from 69 to 213 cases per 100,000 children under the age of 5 years [5–7], but the incidence of KD has also increased in countries worldwide recently. Its most severe complication is the occurrence of coronary artery lesions (CAL), including coronary artery dilatation, artery fistula, myocardial infarction, [8], and coronary artery aneurysm formation [9].

The most common definition of CAL, which can be known as coronary artery abnormality (CAA), is based on the diagnostic criteria determined by the Japanese Ministry of Health: maximum internal diameter > 3 mm in children younger than 5 years old or > 4 mm in children 5 years old and older, the presence of luminal irregularity, or a segmental lumen 1.5 times greater than an adjacent one [10–16]. According to our previous reports, in which we performed a serial analysis of coronary artery lesions (*n* = 341) [17], 35% of KD patients experienced dilatation during the acute phase, 17.2% one month after disease onset, and 10.2% at the two-month follow-up; 4% had persistent CAL for more than one year.

While KD’s clinical features are recognizable, the disease’s underlying immuno-pathogenetic mechanisms are still being investigated, particularly the cause of CAL development. Immune system activation is a key feature of KD, and it is believed that host or infectious pathogenic proteins produced from an unknown focus targeting the endothelial cells of coronary arteries [18, 19]. Consistently, we have also demonstrated toll like receptors 1, 2, 4, 6, 8, and 9, could activate the immunopathogenesis of KD [20]. Persistent monocytosis even after patients are treated with intravenous immunoglobulin (IVIG) has been associated with CAL formation [21], and an increase in eosinophils after IVIG treatment has been inversely correlated with failure rate of IVIG treatment in children with KD [22]. Additional evidences have demonstrated that lower eosinophil levels correlate with lower Th2 cytokines (interleukin-5, IL-5) in KD patients with CAL formation [4, 23].

Practices related to the duration of high-dose aspirin administration vary across medical centers and countries, many of which reduce the aspirin dose once the patient is afebrile, even before administering the IVIG treatment [24]. Upon discontinuing high-dose aspirin, low-dose aspirin (3–5 mg/kg/day) is prescribed until there is no evidence of CAL formation and inflammatory markers have normalized, usually by 6–8 weeks after onset of KD. Patients that develop CAL will continue to receive low-dose aspirin (or other anti-platelets) indefinitely until the inflammatory markers and echocardiography comes within normal range. However, it is believed that anti-inflammatory or immune-modulatory effect of IVIG (2 g/kg) or pulsed methylprednisolone (30 mg/kg, 1–3 days) surpass that of high-dose aspirin and aspirin cannot remove the etiologic substances inducing sustained coronary artery injury [19]. Hsieh et al. [25] reported that high-dose aspirin in acute KD does not influence the response rate to the IVIG therapy, duration of fever, or incidence of CAL. This study reiterates that exposing children to high-dose aspirin therapy in KD is unnecessary. However, no randomized controlled trial regarding high-dose aspirin in acute stage of KD has been available before now. Therefore, we have begun this randomized controlled trial to evaluate the necessity of high-dose aspirin in the acute stage of KD.

## Methods/design

### Study design and medical centers

This trial has been designed as a multi-center, prospective, randomized controlled, evaluator-blinded, non-inferiority trial with two parallel groups to evaluate the effectiveness of IVIG alone for primary treatment of acute-stage KD. The primary endpoint is characterized as CAL formation at 6–8 weeks. Children with KD that meet the eligibility criteria are randomly assigned (1:1) to a test group (that receives only IVIG) or a standard group (that receives IVIG plus high-dose aspirin). This clinical trial is conducted at three medical centers in Taiwan, including:

Site 1: Kaohsiung Chang Gung Memorial Hospital.

Site 2: Linkou Chang Gung Memorial Hospital.

Site 3: Taichung Veterans General Hospital.

### Study objectives

The aim of this randomized-controlled clinical trial is to evaulate whether IVIG alone as the primary therapy in the acute stage of KD is as effective as IVIG combined with high-dose aspirin therapy with regard to inhibiting the development of CAL (with a follow-up of 6–8 weeks). The secondary aim is to compare IVIG resistance rate, duration of fever after IVIG treatment, length of hospitalization, liver enzyme, GI upset symptoms, complete blood count/white blood count, and C reactive peptides between just IVIG and IVIG with high-dose aspirin as the primary therapy in the acute stage of KD.

### Participants

Eligible patients meet all of the following inclusion criteria and none of the exclusion criteria listed:

### Inclusion criteria

Patients eligible for this clinical trial have to comply with all of the requirements below.

1. Male or female, under the age of 18 years old.
2. Fulfilled the AHA criteria for KD as explained below:
3. Fever (more than 38.0 °C ear temperature) > or = 5 days, as well as 4 of the 5 following symptoms
4. Diffuse mucosal inflammation (strawberry tongue, dry and cracked lips)
5. Bilateral non-purulent conjunctivitis
6. Dysmorphous skin rashes
7. Indurative edematous change over the hands and feet, or desquamation over the fingertips or toes
8. Cervical lymphadenopathy (one or more nodule at least 1.5 cm in diameter)
9. An informed consent form (ICF, appendix B) signed by the patient or a legal guardian.

### Study intervention

Patients that had any of the conditions described below are not eligible for this clinical trial. All patients will receive IVIG (2 g/kg) over 12 h along with (group 1) or without (group 2) high-dose aspirin. After the fever subsides, low-dose aspirin (3-5 mg/kg/day) will be prescribed until 6–8 weeks, as suggested by the American Heart Association guidelines [9]. All the patients in the study group receive IVIG (2 g/kg) over 12 h plus high-dose aspirin (80–100 mg/kg/day) until the fever subsides. Once the fever subsides, low-dose aspirin (3–5 mg/kg/day) will be prescribed until 6–8 weeks after the disease onset. The researchers will try to minimize the concomitant treatments for the patients during the study. Any concomitant treatment must be recorded on the CRF. The use of steroids, anti-TNF, anti-IL6, anti-IL17, anti-CD20 (and other biologic agents) is prohibited during the study treatment.

### Exclusion criteria

Patients that fulfill any of the following criteria cannot be included in this clinical trial.

1. Had symptoms that did not completely match the KD criteria.
2. Had an acute fever for < 5 days and > 10 days
3. IVIG treatment at another hospital before being referred to the study center.
4. Treatment with corticosteroids, other than the inhaled form, in the two weeks prior to joining the study;
5. The presence of a disease known to mimic Kawasaki disease (such as adenovirus infection, toxic shock syndrome etc.).
6. Previous KD diagnosis.
7. Inability to take aspirin (such as history of hypersensitivity to aspirin, G6PD deficiency, recent herpes zoster infection or vaccination, etc.)
8. Afebrile prior to enrollment
9. Severe concomitant medical disorders (e.g., immunodeficiency, congenital heart diseases, chromosomal anomalies, metabolic diseases, collagen diseases, nephritis, etc.)
10. Suspected to have an infectious disease, including sepsis, septic meningitis, peritonitis, bacterial pneumonia, varicella, and influenza
11. Judged by the researcher to be unsuitable for this trial.

### Withdrawal criteria

Participants will be withdrawn from the clinical trial if any of the conditions stipulated below occurs. If a patient has to be withdrawn from the study, the reason is to be recorded in the case report form (Supplementary1.), as well as the participant’s medical record.

1. The participant or his/her legal guardian decides to withdraw his/her informed consent.
2. The participant is lost for follow-up.
3. The researcher considers the participant to no longer be physically and/or psychologically fit to remain in the study.
4. The participant develops an adverse event (AE) such that the researcher considers stopping the study treatment necessary.

### Assessment outcomes and variables

All participants will be given a structured questionnaire in order to collect demographic data, such as age, gender, and ethnicity. We record body temperature every 6 h during the febrile stage. The primary outcome is CAL, which is described as the luminal diameter of more than 3.0 mm in a child under the age of 5 years old or more than 4.0 mm in those aged 5 years and older, when the internal diameter of a segment is 1.5 times or greater than that of an adjacent segment, or when the luminal contour is clearly irregular or has a Z score > 2.5 SD [22, 23]. We estimate the Z score of the proximal right coronary artery, left main coronary artery, and proximal left anterior descending artery, as well as the maximum Z score of coronary arteries at baseline, weeks 1, 2, 4, and 6–8 with 2D echocardiography. The body weight and height used to calculate Z score come from the website of the Taiwan Society of Pediatric Cardiology (http://www.tspc.org.tw/service/z_score.asp). The secondary outcome is IVIG resistance, which is described as persistent or recrudescent fever at least 48 h but no more than 7 days after completing the first IVIG treatment [26].

### Laboratory variables

Blood samples collected for complete blood count, aspartate transaminase (AST), alanine transaminase (ALT), and CRP are checked at baseline, weeks 1, 2, 4, and 6–8. If the concentration of CRP is undetectable, we credit it as 50% of the lower limit of the assay.

### Adverse events (AE) and serious adverse events (SAE)

We record AE and SAE, and the severity of AE is based on Common Terminology Criteria for Adverse Events (CTCAE) version 4.03 (Appendix A).

### Sample size

The sample size determination is based on the results of the retrospective data (24). The estimated proportions of CAL are 17% after IVIG treatment combined with high-dose aspirin and 15.4% after IVIG alone. We consider a difference less than 10% as having no clinical significance. The following contents are the statistical basis for estimating sample size in this trial:

$$ {\mathrm{H}}_0:{\mathrm{P}}_{\mathrm{T}}-{\mathrm{P}}_{\mathrm{S}}\leqq \updelta $$

$$ {\mathrm{H}}_{\mathrm{A}}:{\mathrm{P}}_{\mathrm{T}}-{\mathrm{P}}_{\mathrm{S}}>\updelta $$

P_T_: CAL-free rate in test group =0.846.

P_S_: CAL-free rate in standard group =0.83.

δ = −0.1.

$$ n=\frac{{\left({Z}_{\alpha }+{Z}_{\beta}\right)}^2}{{\left(\varepsilon -\delta \right)}^2}\left[{P}_T\left(1-{P}_T\right)+{P}_S\left(1-{P}_S\right)\right] $$

Assuming that the type I and type II error rates are α = 0.05 and β = 0.2, respectively, the clinically meaningful difference δ is − 0.1. The CAL-free rate of the test group (IVIG alone) P_T_ is 0.846 while that of the standard group (IVIG + high dose aspirin) P_S_ is 0.83. Define *ε* = *P*_*T*_ − *P*_*S*_. Assuming the sample sizes of the two groups are equal, each group for the non-inferiority trial must have 125 patients. In order to offset a maximum dropout of 10%, 139 patients are needed for each group. Therefore, a total sample size of 278 patients is required for this trial.

### Allocation

The eligible participants will be randomized to either the IVIG treatment alone or the IVIG plus high-dose aspirin treatment at an equal ratio. The researcher or his/her delegate will contact the Clinical Trial Center of Kaohsiung Chang Gung Memorial Hospital (KCGMH- CTC) once the patients have been confirmed to fulfill all the inclusion/exclusion criteria. The CTC-KCGMH will assign a treatment arm to the patient. Randomization will be categorized by participating institutions using permuted blocks of random sizes, which are not to be disclosed, thus ensuring concealment.

### Blinding

Both participants and the study’s researchers are unblinded to the IVIG alone group or the IVIG plus high-dose aspirin group. The primary endpoint of CAL will be determined by two pediatric cardiologists blinded to the assigned treatment group.

### Data collection methods and management

All data, including demographic data, medical history, medical records, laboratory data, AE, and SAE, is recorded at CRF, and the researchers will maintain individual records (ICF and CRF) for each patient as source data. Each CRF will be copied and sent to KCGMH-CTC for data management. The follow up 2D echocardiograms at weeks 1 (Day 7 ± 1), 2 (Day 14 ± 2), 4 (Day 28 ± 4), and 6–8 (Day 49 ± 10) shall be digitally recorded at the respective institutes and interpreted at a core laboratory by two pediatric cardiologists blinded to patient identity and group assignment.

### Statistical methods

All of the baseline characteristics are used to study the comparability of both trial arms. The primary outcome evaluation and safety evaluation are carried out on the intention-to-treat (ITT) population. Continuous variables will be described using either the mean (standard deviation) or median (interquartile range), as appropriate, and categorical variables will use count (percentage). Participants’ characteristics are compared using a χ2 test for categorical variables and a t-test or Wilcoxon rank sum test for continuous variables. *P*-values< 0.05 are considered statistically significant.

## Discussion

Aspirin has been used to treat KD for years, even before the treatment of IVIG [27]. In the acute stage of KD, aspirin is administered at 80 to 100 mg/kg per day (30–50 mg/kg in Japan) [25] with IVIG administration. Even though high-dose aspirin has significant anti-inflammatory and anti-platelet functions, it does not lower the incidence of CAL formation. Saulsbury et al. were the first to report that comparisons of two dosages of aspirin plus IVIG (2 g/kg) revealed no benefit in high-dose aspirin compared to low-dose aspirin in treating the acute stage of KD [28]. In a previous study, we examine 851 KD patients from two medical centers in Taiwan (Chang Gung Memorial Hospital-Kaohsiung and Kaohsiung Veterans General Hospital) [29]. The patients are divided into Group 1, with high-dose aspirin (*n* = 305) and Group 2, without high-dose aspirin (*n* = 546). No significant differences were found between Groups 1 and 2 with regard to gender (*p* = 0.51), IVIG resistance rate (31/305 vs. 38/546, *p* = 0.07), CAL formation rate (52/305 vs. 84/546, *p* = 0.67), or total length of hospital stay (6.3 ± 0.2 days vs. 6.7 ± 0.2 days, *p* = 0.13). These results indicate that high-dose aspirin in acute KD does not affect disease outcomes. Therefore, administering high-dose aspirin to treat KD appears to be unnecessary [29]. Furthermore, high-dose aspirin decreased hemoglobin levels and affected the decrease of inflammation markers following IVIG administration [29, 30]. A systemic meta-analysis also reported that both low-dose aspirin and high-dose aspirin have a similar incidence of CAL development in KD patients [31]. All of these findings suggest that high-dose aspirin treatment may not be correlated with higher benefits with regard to CAL formation, IVIG resistance, and shorter hospital stays.

In conclusion, various retrospective studies have shown that high-dose aspirin does not offer any significant benefits in the acute stage of KD. However, no randomized controlled trial regarding the use of high-dose aspirin in the acute stage of KD has yet been done. Therefore, we have initiated this randomized controlled trial to determine whether high-dose aspirin is actually necessary for treating the acute stage of KD.

### Trial status

This trial is currently in the participant recruitment phase.

## Abbreviations

AE: Adverse event
CAL: Coronary artery lesion
CRF: Case report form
CRP: C reactive peptide
IVIG: Intravenous immunoglobulin
KD: Kawasaki disease
SAE: Severe adverse event
